# Non-innocent partners: bias distribution between photoanodes and cathodes in photoelectrochemical overall water-splitting cells

**DOI:** 10.1093/nsr/nwae155

**Published:** 2024-05-01

**Authors:** Wei Li, Dunwei Wang

**Affiliations:** Department of Chemistry, Merkert Chemistry Center, Boston College, USA; Department of Chemistry, Merkert Chemistry Center, Boston College, USA

Photoelectrochemical (PEC) overall water splitting (OWS) represents a highly promising strategy to directly harvest solar energy and store it in the form of fuels, offering unique benefits such as mitigating issues connected to the intermittent nature of sunlight. The electrical voltage required for OWS may be, in principle, separated into different components: the thermodynamic driving force of generating oxygen and hydrogen at standard temperature and pressure (*E*^0^_cathode_ − *E*^0^_anode_ = −1.23 V), the overpotentials necessitated to surmount the kinetic barriers for the oxygen evolution reaction (OER) and hydrogen evolution reaction (HER) at practically meaningful current densities and the losses due to ohmic resistance within the cell.

Given that water splitting involves two fairly well-defined half redox reactions, separate investigations of the photoanode for OER and the photocathode for HER are logical but challenging. Experimentally, a three-electrode configuration is typically employed to focus on the intrinsic efficiency of a given (photo)electrode at a time, so as to exclude the parasitic losses due to the various components, especially those associated with the counter reactions. This is achieved by measuring the current between the working (photo)electrode and a counter one relative to a reference electrode with known (and stable) standard potentials [1]. To see the utility of such a treatment, let us consider a simplified discussion as follows. OERs involve four protons and four electrons, and they tend to be inherently sluggish, posing a significant challenge to practical OWS. By comparison, HERs are typically considered to be faster and, therefore, are expected to be technically less challenging for practical OWS. Even for more complex reduction processes such as CO_2_ reduction, the penalty one pays at the (photo)cathode is expected to be less than that at the (photo)anode. Indeed, it was suggested by using thermodynamic analyses that 90% of electricity input is consumed by OER when coupling with CO_2_ reduction [2]. In response to such an insight, extensive efforts have been directed toward optimizing the photoanode materials to provide greater photovoltages and developing more efficient catalysts to reduce the kinetic overpotentials. An alternative approach to reduce the overall bias consumption by the (photo)anode is to replace OER with kinetically and thermodynamically more favorable oxidation half reactions such as glucose oxidation [3]. This progress notwithstanding, the actual bias distribution between photoanodes and cathodes in a PEC-type overall reaction cell has not been experimentally implemented. Furthermore, the synergistic interplay between the OER at the anode and the HER at the cathode remains poorly understood.

Dang *et al.* recently developed an experimental technique to respond to this challenge and fill in a critical knowledge gap [4]. They measured bias distribution in a two-electrode PEC OWS cell with representative photoanodes and Pt metal as the cathode. Steady current density was quantified and the photoanode/cathode potentials along with the corresponding partial voltage ratio of the photoanode were recorded against additional sensing electrodes as a function of the overall cell voltage. For photoanodes with low photovoltages (*V*_ph_ of Ni/n-Si, α-Fe_2_O_3_ < 1 V), the bias distribution profiles revealed three distinct regimes: between 0 and 0.4 V_cell_, a decent amount of the applied bias was used to charge the photoanode (>50% for Ni/n-Si and >16% for α-Fe_2_O_3_); between 0.4 V_cell_ and the voltage at which the photocurrent started, nearly 100% of the increased bias was used to charge the cathode; as the bias increased to the point of photocurrent saturation, the majority of bias remained on the cathode to drive HER. On the contrary, for photoanodes with high photovoltages (*V*_ph_ of BiVO_4_, TiO_2_ > 1 V), only two regimes were observed, with bias consumption for HER thermodynamics on the cathode evident at lower biases (0–0.3 or 0.4 V_cell_). The findings in this work are surprising, as the results suggest that HER could also be a limiting factor for OWS in a two-electrode cell, particularly for photoanodes with low photovoltages. This necessitates the exploration of various reductive half reactions to manage bias consumption. The authors successfully achieved an unbiased PEC cell by substituting HER with the Fe^3+^ reduction reaction (FRR) using a Ni/n-Si photoanode and a Pt cathode. It is, nevertheless, important to note that the measured voltage across the working electrode and the reference electrode, which indicates the bias distribution on the photoanode (or cathode), is also influenced by the distance between the two electrodes due to the ohmic resistance in the electrolyte. Additionally, the physical meanings behind selecting the specific value of 0.4 V_cell_ as the regime boundary are intriguing and worth additional studies.

The authors further demonstrated that bias consumption can be regulated through tuning the electrolyte pH. The results showed that the photocurrent decreased and the bias distribution profiles of both electrodes shifted positively with a decrease in the OH^−^ concentrations. Lastly, it is also worth noting that the potential drop resulting from the decrease of ionic conductivity in electrolyte may be yet another study point for future research to address. The authors also attempted to lower the bias consumption on the cathode by using an acidic electrolyte in a bipolar membrane-based H-cell. Consequently, the bias distribution on the photoanode increased, accompanied by increasing photocurrents. It would be interesting to measure the potential losses due to the pH gradient under appreciable current densities [5].

In essence, the presented work established an experimental method to measure the actual bias distribution in a two-electrode PEC-type overall reaction cell, while also devising an unbiased PEC overall reaction cell through alternative redox reactions based on generated knowledge. This study offers insights for system-level cell design optimization and paves the way for practical applications of PEC OWS.
